# Teachers’ perspective on implementation of menstrual hygiene management and puberty education in a pilot study in Bangladeshi schools

**DOI:** 10.1080/16549716.2021.1955492

**Published:** 2021-08-02

**Authors:** Mehjabin Tishan Mahfuz, Farhana Sultana, Erin C. Hunter, Farjana Jahan, Farhana Akand, Shifat Khan, Mosammat Mobashhara, Mahbubur Rahman, Mahbub-Ul Alam, Leanne Unicomb, Stephen P. Luby, Peter J. Winch

**Affiliations:** a Environmental Interventions Unit, Infectious Disease Division, International Centre for Diarrheal Disease Research Bangladesh (icddr,b), Bangladesh; b School of Public Health, Faculty of Medicine and Health, University of Sydney, Sydney, Australia; c Woods Institute, Stanford University, Stanford, CA, USA; d John Hopkins Bloomberg School of Public Health, Baltimore, MD, USA

**Keywords:** Menstrual hygiene management (MHM), menstrual health, social and behavior change communication (SBCC), education, education barriers

## Abstract

**Objective:**

To assess the perspectives of Bangladeshi teachers on the feasibility of delivery and potential for long-term sustainability of puberty and menstruation education in urban and rural schools.

**Method:**

We developed a multi-module puberty and menstrual hygiene management education curriculum that teachers piloted for six months in four urban and rural government and private schools in Bangladesh. We conducted monthly assessments during piloting, discussion for manual revision and four group discussions with 20 participating teachers to understand perceived benefits, barriers, and sustainability of puberty and menstruation education among school children.

**Results:**

Teachers acknowledged the importance of school-based puberty and menstruation education to improve students’ perception and preparedness. They found that the training and instructors’ manual they received were useful tools for effectively communicating with students. Teachers noted school and community pressure to de-emphasize educational content not included on nationally standardized examinations, and insufficient time and pre-service training for teaching sensitive topics served as barriers to implementing the pilot curriculum.

**Conclusion:**

Pressure from school authorities and community may hinder the successful long-term delivery of school-based puberty and menstruation education programs that are external to the national curriculum. Our findings indicate that feasibly and sustainably improving education on these topics in Bangladeshi classrooms should be achieved through 1) revision of the current national curriculum to incorporate more comprehensive puberty and menstruation information including its physiology, management, and social context, 2) adequate training and support for teachers to deliver the content, and 3) incorporation of puberty and menstruation content into students’ national examinations which may better ensure teachers are given the tools and opportunity to prioritize teaching this content.

## Background

To have positive menstrual experiences and attain optimal menstrual health, girls require more than just affordable and safe menstrual materials and access to sanitation, washing, and waste disposal facilities. They also require accurate and timely knowledge about menstruation from informed professionals, referral and access to quality health services, and positive social norms [1]. Yet in many low- and middle-income countries, girls face barriers in addressing their menstrual needs – particularly stemming from pervasive menstrual stigma and limited access to accurate and timely information about menstruation [2–7]. Research across diverse contexts including Bangladesh, Tanzania, and Ghana, among others have explored how socio-cultural and religious proscriptions that limit open discussion of menstruation leave girls unprepared for the physical and psychological transformation of puberty and menstruation [4,5]. A meta-synthesis of 76 studies across 35 low- and middle-income countries found that girls who reach menarche without prior knowledge of menstruation commonly describe feeling distressed, ashamed, and may delay seeking help due to fear of punishment [5].

Limited access to puberty information and school environments that are not supportive of the menstruation-related needs of students may hamper girls’ school attendance and academic performance, contributing to attrition that can result in early marriage, adolescent early pregnancy and multiple associated negative health and economic impacts [8–12]. Schools play an important role in disseminating health information where a large number of children are easily reached at once [13,14]. In recent years, menstrual hygiene management programs have been implemented in schools in many LMICs, predominantly driven by the water, sanitation, and hygiene (WASH) sector [15]. Such programs have often relied on project staff or health professionals hired to implement menstruation education to adolescent girls [16–18] but there is limited literature exploring schoolteachers’ experiences as implementers in LMICs. Sharing context-specific implementation lessons learned from the piloting of school-based programs that engage teachers can provide improved understanding of how best to integrate menstrual hygiene and health into education systems for better sustainability [19].

We developed and piloted a multi-component intervention intended to foster supportive school environments for menstruating students in Bangladesh (main intervention outcomes reported separately). The aim of this paper is to assess the teachers’ perspectives on the feasibility of delivering the puberty and menstruation education sessions in schools which were part of the broader pilot intervention. We also explored the potential for their long-term sustainability in primary and secondary schools in urban and rural Bangladesh.

## Methods

### School selection

**Figure 1.**
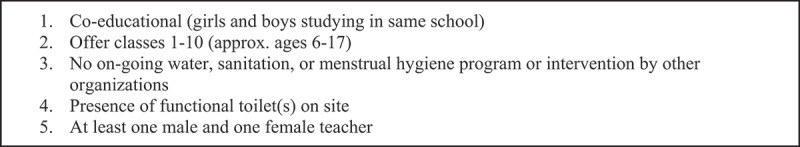
Inclusion criteria for school selection

The research presented in this paper was conducted in four schools in Dhaka Division, Bangladesh (two urban, two rural), among which one urban school was public and the rest were private schools. Our study team collected a list of schools in urban Dhaka from the Divisional Education Department and in rural Manikganj District from the office of sub-district level Administrative Education Officers. Team members communicated with 200 schools via telephone and identified 40 schools that met our inclusion criteria (Figure 1). We visited 20 randomly selected schools out of the 40, and purposively selected eight schools (four for an earlier formative research phase, four for the intervention piloting phase on which we report in this paper) based on head teachers’ willingness to participate. We obtained permission to conduct research from the Dhaka Zonal Office, Directorate of Secondary and Higher Education; the Dhaka Divisional Office, Directorate of Primary Education; and School Management Committees. Participating schools ranged in size from 900 to 2100 students, and students enrolled in classes 5–10 (ages 9–17) participated in the pilot intervention. The education curriculum and teacher training presented in this paper was one component of a broader pilot intervention package that including engagement with school administration and parents, established school gender committees, installed waste disposal systems in girls’ toilets [20], and provided students with menstrual hygiene packs consisting of cloth pads, undergarments, carrying bags and menstrual calendar. The schools were also provided with a stock of disposable pads for girls to access upon request.

### Description of curriculum and teacher training

The education curriculum aimed to empower students with improved knowledge, skills, and self-efficacy to manage changes during puberty and support girls to more easily address their menstrual needs so that they did not have to miss school during menstruation. We designed the education curriculum following a formative study with students and teachers and an intervention development workshop with government and relevant non-government stakeholders to understand the gaps in provision of puberty and menstrual hygiene management information at schools. The curriculum for both girls and boys covered the physical, mental, and emotional changes that occur during puberty; male and female reproductive systems; menstruation, menstrual hygiene management, and overcoming menstrual stigma; and nutrition. Additionally, girls received a session on practical methods of managing menstruation and menstrual symptoms while boys received a life skills session on problem-solving (Table 1). The sex-separated intervention delivery was recommended in the formative study by students and teachers and was feasible to implement due to the current sex-separated class structure in the study schools.

**Table 1.** Module overview of piloted puberty and menstruation education curriculum in urban and rural schools of Bangladesh in 2017–2018

Module and Session No.	Topic	Contents
Module 1	Growing Up	This module provides an introduction to the physical and mental changes that girls and boys can expect to experience as they move through adolescence and puberty.
Session 1.1	Introduction to puberty	
Session 1.2	Physical changes in boys	
Session 1.3	Physical changes in girls	
Session 1.4	Mental and emotional changes	
Module 2	Reproductive Systems	This module covers the basics of the anatomy of the male and female reproductive systems and provides information about nocturnal emission.
Session 2.1	Male reproductive system	
Session 2.2	Nocturnal emission (Wet dreams)	
Session 2.3	Female reproductive system	
Module 3	Menstruation	This module introduces menstruation to both boys and girls. Girls receive detailed information about how to address menstrual needs including accessing and using hygienic menstrual materials, mitigating menstrual symptoms, and seeking support. Boys receive information on how they can support menstruating peers and learn about positive approaches to problem solving.
Session 3.1	Menstrual cycle	
Session 3.2	Menstrual hygiene management: Part 1 (Girls Version) Supporting Our Peers (Boys Version)	
Session 3.3	Menstrual hygiene management: Part 2 (Girls Version) Problem-Solving (Boys Version)	
Session 3.4	Overcoming stigma	
Module 4	Nutrition	The last module provides basic information about nutrition requirements during adolescence for growth and development and the importance of eating a diet high in iron and vitamin C for menstruating girls.
Session 4.1	Nutrition for growing adolescents	

The curriculum comprised four modules of 1–4 sessions each and the entire curriculum could be completed in a total of 190 minutes over a six-month period. Sessions began with a motivating game aimed to draw students’ attention and ensure a smooth segue into the sensitive discussion, followed by delivery of core content using visual aids and practice activities, and concluded with a period of question and answer. In anticipation of inhibitions around teaching sensitive content, the teachers’ manual contained short scripts that teachers could deliver verbatim to their students to help them deliver the sessions more comfortably if they chose, although they were not limited to using the scripts.

The current national textbooks for classes 6–10 (age 10–16 years) contain limited information on menstruation and puberty that is scattered throughout different subjects and there is no information regarding the practical aspects of menstrual management. The curriculum developed for this study expanded upon the content from the textbooks, arranged the material in a progressive and organized manner, and incorporated participatory activities. It was intended to provide opportunities for girls to build their confidence in addressing their menstrual needs, thus self-efficacy theory guided the design of sessions [21]. We created opportunities for girls to observe others performing menstrual care tasks (e.g. through images, video, mock demonstrations by teachers and classmates), allowed time for girls to practice themselves (e.g. practice using a menstrual calendar and role playing how to ask for menstrual materials), and encouraged teachers to provide positive verbal feedback and encouragement to students as they learned and to facilitate friendly environments during class sessions.

Head teachers of the four intervention schools nominated male and female teachers to receive training to deliver the curriculum in their schools. A total of 20 teachers (11 female and 9 male) participated in the study. Among the female teachers, one was a head teacher and the rest were home science teachers. The male teachers included three head teachers and the rest were physical education teachers.

Existing textbooks for home science and physical education included chapters on menstruation and puberty, so these teachers were nominated as most appropriate to administer the new pilot curriculum. We provided an initial two-day training and a two-day refresher training halfway through the piloting period. Teachers were trained both on curriculum content and techniques for delivery using a comprehensive teachers’ manual. During training, teachers read and familiarized themselves with the content and instructions of each session in the manual and asked clarification questions. They then participated as ‘students’ while trainers delivered the session; then teachers volunteered to practice delivering the session to each other. Training on each session concluded with a discussion period to debrief on the experience and make modifications to lesson plans. We provided teachers with visual aids in the form of locally illustrated flipcharts and electronic slide decks as well as all materials needed for classroom games and activities. At the end of training, teachers developed a tentative schedule for completing the sessions in their school. Most schools planned to conduct short sessions weekly over the six-month piloting period. Female teachers provided sessions to schoolgirls and male teachers provided sessions to schoolboys separately.

### Data collection and analysis

We gathered feedback from teachers using three methods. First, we spoke to teachers during monthly assessments that our study staff conducted throughout the intervention piloting period. Each month, a study team member observed two teachers’ classroom sessions in each school (total of 12 observations from all schools over six months), and then immediately had a short, informal debriefing discussion with teachers (approximately 10 minutes) to note their reflections on barriers, facilitators, students’ attitudes, and components of the session that were easy and difficult to deliver. After completion of the piloting period, study staff conducted one meeting in each school (4 total) with the male and female teachers to take their suggestions for revising the teachers’ manual. The interviewers had a list of guiding topics to steer the discussions. The research team took detailed field notes during meetings that lasted 30 minutes on average. Lastly, we conducted one focus group with teachers in each school (4 total) using a semi-structured topic guide to explore their experiences with the entire intervention package. Focus groups comprised 3 to 8 participants each (including male and female teachers), lasted an average of 40 minutes, and were audio recorded with permission of participants. Interview staff were male and female researchers with social science backgrounds and experience in qualitative research.

A study team member transcribed the audio recordings from Bengali directly into English for analysis. The first author (MTM) familiarized herself with the transcripts and then performed initial coding of the data both inductively and deductively using *a priori* codes. MTM then categorized the codes with discussion from the broader team as follows: benefits and drawbacks of intervention components, facilitators, and barriers to conducting education sessions, recommendations for improvement, and how this intervention can be nationally scaled up and sustained.

## Results

### Perceived benefits of teachers training and education sessions

The teachers contended that delivering content about puberty and menstruation in schools is important but admitted feeling uncomfortable during class sessions despite training. The teachers mentioned that students, teachers, parents, and head teachers were unaccustomed to discussion on menstruation and puberty due to social and cultural proscriptions. The teachers also mentioned that they were not adequately prepared with information or training to teach students on these topics before the project.

*Teachers’ training* [from the project] *was good. We* [previously] *did not have any idea how to deal with students with this sensitive issue, how to discuss about puberty with them. The training duration was perfect, as the content was not massive*

-Male teacher, urban school

Teacher reported that having the scripts in their teachers’ manual that could be read aloud verbatim prepared them for delivery and helped them ease into the sessions more confidently, especially in the beginning of the intervention. After the piloting period, some teachers suggested longer training for better understanding of reproductive physiology.

*Maybe, duration of training can be increased, one or two days more. The training sessions were like a rushing train! But, topics and discussion were vast. Personally, my knowledge was increased; even I did not know many details. Those who came from science background know much about physiology. But those who studied commerce or humanities, lack this* [basic] *knowledge! Personally, I am benefitted!*

-Female teacher, urban school

The teachers reported noticeable change in the school environment and girls’ behavior after teachers’ training and implementation of the education sessions. Teachers described girls as more open and comfortable in discussing menstruation with teachers, and girls even asked for menstrual absorbents from male teachers. Teachers discussed how during parent-teacher association meetings guardians reported seeing a ‘positive’ change in girls’ behavior regarding menstrual care.

*I have seen one girl in class VI who menstruated suddenly in school, and she came to take a pad during tiffin* [lunch] *break. One Gender Committee girl member from class 10 counseled her, “There is nothing to worry, you will use these pads, fold it in this way and fix it with underwear,” she demonstrated it with a friendly attitude!*

-Female Teacher, urban school

Guardians spoke during school management committee meetings. Some had negative comments. Most were positive … One guardian said, ‘I have never asked my daughter about these; she is in class 6. But, I can feel this change. Now, she openly spreads and dries her menstrual cloth. If she did not attend the classes, she would do it secretly.’

-Female teacher, urban school

### Barriers to conducting education sessions

Most teachers noted that time management in the busy school routine was a major barrier to conducting the sessions in a timely manner. Almost all teachers were unable to conduct the motivating games from the manual due to large classes consisting of at least 40 students and small classroom space. Although the study team and teachers had co-developed a session delivery schedule before piloting began, the teachers were unable to accommodate all the sessions.

… I am in a rush to make up the syllabus before the scheduled annual exam starts. Hence, I could not deliver classes in the last few days. In every class, new students will be admitted. These new girls get surprised, “How do you know all this?” I want to deliver additional classes, but time does not permit it! We have regular class routine. If it is integrated with the existing curriculum, then time will not be a problem.

-Female teacher, urban school

They reported that besides teaching, the government teachers are assigned multiple civic responsibilities, such as monitoring during election duties, board or state entrance exams, and other local government events, which is an added burden on their schedule. Though the study team had previously met with head teachers and school committee members who agreed to support the intervention components, the teachers ultimately reported reluctance from the school management committees and parents to take school time to provide sessions on which the students will not be evaluated by national exams.

If these topics are included in existing syllabus, then, we will not need additional time to conduct these classes. But, in the present system it was hectic. For class 8, we get only six to seven months to complete the syllabus; there are many examinations, term, pre-test, and test and model test. We are always in a rush to catch up with the schedules.

-Female teacher, rural school

### Potential for sustainability

Most teachers expressed that to ensure long-term inclusion of menstruation and puberty related training for students it should be incorporated in the national education curriculum. Other recommendations for sustainability included training younger teachers to establish a more friendly communication between teachers and students:

*If there are two sections* [of the same class to accommodate large number of students], *at least four teachers should be involved, especially young teachers aged between 35 to 40 years.*

-Female teacher, urban school

Some teachers mentioned that the sessions can be sustained in individual schools if the head teachers actively support teachers by regularly providing them extra class time and schedule in the routine as they did during the intervention period. The teachers conducted sessions during class time for the course of the intervention, though they have mentioned that this practice will not continue after the intervention period. Teachers mentioned that sessions on puberty and menstruation should be delivered by teachers, as they believe that students are more attentive than with other mediums of communication, such as internet or television.

*Here* [in this study], *the students were under a system, they learnt through a system. When teachers will discuss about these changes, the students listen to them attentively, they take the classes seriously. It is not possible using other media.*

-Female teacher, urban school

The teachers agreed that extracurricular education programs are successful as long as the organizing institutions, generally non-government organizations (NGOs) are overseeing the activities and providing incentives. These programs are usually for a short duration and the teachers are not obligated to continue teaching students if it is not part of the government program.

*If this topic is included in the existing curriculum* [for evaluation on national exams], *it will be good. We have scheduled class routine. We had to manage extra time to conduct these* [extracurricular] *classes*.

-Female teacher, rural school

*If the head teachers permit, we can arrange session for girls, either in science or home science classes*.

-Female teacher, urban school

## Discussion

The Government of Bangladesh has progressively recognized the importance of sexual and reproductive health rights education in the school system. Bangladeshi women’s age at first marriage has increased from 14.3 to 15.7 years over a decade, and post-primary education attainment has increased 1.4 years on average from 1993 to 2011 [11]. Recent programs undertaken by the government aim to prevent gender-based violence and meet sexual and reproductive health rights needs of adolescents in Bangladesh [22]. However, the prevalence of child marriage and girls’ lack of knowledge about menstruation in Bangladesh is still high compared with other countries [11].

Education on puberty and menstruation is inconsistently implemented in Bangladeshi schools. Considering that only about 12.6% of girls aged 9–13 are out of school in Bangladesh, adequate information through schools is an efficient means to knowledge dissemination (cite). A prior study in rural Bangladesh showed that providing school-based menstruation education improved girls’ self-reported school attendance during menstruation from baseline [23]. However, the education sessions were provided by research assistants hired for the project and the study authors concluded that communication needed to be improved between female students and their teachers [23]. Our study extends the state of knowledge in this area in that we trained existing schoolteachers to provide comprehensive puberty and menstruation education sessions to their students – which may be a more sustainable approach to scale up. Teachers in our study agreed on the importance of teaching menstrual hygiene management and puberty education to students. They perceived that the education sessions made students more open to discuss puberty-related topics with their teachers and peers. We see this as an additional benefit of the education being provided by schoolteachers – it provides opportunities for students to increase their knowledge while also providing opportunities to improve communication about menstrual needs between teachers and students.

Teachers recommended incorporating the comprehensive information about puberty and menstruation piloted in our study into the Bangladesh national curriculum and including the content on students’ standard examinations because doing so would protect time to enable them to teach the material. Bangladeshi schools are categorized based on students’ performance in two major curriculum-based board examinations, which is a barrier to allowing extracurricular activities during school hours. A higher score in the board examination increases the chances of a student’s enrollment to a competitive university and increases the school’s ranking, resulting in more admissions [24]. The contents of the examinations are derived from selected chapters of the textbooks, which leads teachers, school authority, and parents to emphasize those topics and deemphasize or skip entirely those that do not appear on examinations. The existing chapters on menstruation and puberty in the standard textbooks are not currently included in the board examinations, and thus heavily deprioritized. Even though teachers in our study saw value in teaching students about menstruation – and recognized positive changes in their students and the school environment during the pilot – the pressure to cover material that will be examined made them feel powerless to sustain the sessions unless the content was formally incorporated into the standard curriculum and exams.

Teachers must have adequate mastery of the subject matter to develop their own strategies to connect with students to create a positive impact [25]. Consistent with other study findings in both LMICs and high-income countries, our study participants mentioned that the training and experience they gained through the project increased their level of confidence in providing puberty and menstruation education; and this has played an important role in their interest and motivation [26,27]. However, some key challenges arose during our pilot that should be considered in future programming. Most teachers entered the project’s training with very minimal knowledge of reproductive anatomy and physiology, yet our training model assumed teachers would have a basic level of understanding at baseline. Thus, training did not allow sufficient time for all teachers to fully grasp the new material, as the primary focus was instead on how to deliver the content in engaging ways. The traditional pedagogical approach in our study context is one-way and the teachers had difficulty adapting to the interactive mode of session delivery in the intervention. The teachers’ lack of familiarity with an interactive teaching approach could be another underlying reason why they skipped the motivating games that were meant to open each session.

Teachers universally reported entering the project feeling underprepared to deliver sensitive information due to lack of adequate training and lack of proper vocabulary to start and facilitate the conversation with students. This is consistent with literature from other resource-constrained settings that have shown common barriers for teachers including large classes and limited training and education backgrounds that inadequately prepare teachers for delivering information to cater to diverse needs of students (Table 2)[28–31]. Strengthened pre-service teachers’ training programs that better prepare teachers to deliver lessons on sensitive topics could help promote supportive school environments for menstruating students and facilitate more active involvement of teachers [32]. Incorporating practice sessions and role-playing in the training to prepare teachers for delivering sensitive information to large groups of students in a classroom setting could facilitate breaking social barriers. It is also important that such training allows schoolteachers time to think deeply in conversations with each other, reflecting on their own constructions of menstruation and how such constructions influence the way they approach teaching on the topic. Research in New Zealand found that teachers constructed menstruation as something embarrassing although their students considered learning about menstruation to be important and found interactive portions of menstrual education sessions to be fun [33].

**Table 2.** Global comparison for teachers’ barriers in conducting classroom sessions on puberty, menstruation, and sexual and reproductive health education

	USA [34,35]	England [36]	Australia [37]	Swaziland [25]	Uganda [5]	Peru [38]	Thailand [39]	Malaysia [40]
Time management	✓	✓		✓	✓	✓		
Large classroom				✓	✓			
Lack of state priority	✓	✓	✓	✓	✓		✓	✓
Lack of teachers’ training		✓	✓	✓	✓	✓	✓	✓
Lack of support	✓			✓	✓	✓	✓	✓
Social opposition	✓			✓	✓			✓

Since our study was focused on the piloting of an intervention, we worked in a small number of purposively selected schools based on our inclusion criteria and head teachers’ willingness to participate in the study; therefore findings may not represent the country scenario broadly. Conclusions from our Study are limited in that we did not include religious schools (*Madrasas*) in the study. Madrasa education in Bangladesh is controlled by the Madrasa Education Board, a branch of Ministry of Education. The Madrasa curriculum generally aligns with national education curriculum and contains the same books and information on puberty and menstruation with more emphasis on religious studies [22]. In 2016, about 30% of total students were enrolled in Madrasa. Among them, over 50% were girls [22,41]. Further research on perceptions and practices in these more conservative contexts may provide useful insights for future policy and administrative planning. Teachers in our study reported hearing both positive and negative reactions from parents of the students who received the pilot intervention though the specific feedback from parents and community have not been explored. Future research should explore the role of parental engagement in the sustainability of school-based puberty and menstruation education in this context.

## Conclusion

Our study demonstrated that Bangladeshi schoolteachers who received training and piloted a comprehensive puberty and menstruation education curriculum with their students found value in teaching the material and felt it was important to continue providing such content to future cohorts of students. However, their own minimal baseline knowledge on the topics and limited pre-service training on teaching sensitive material negatively impacts their confidence in delivery. This paper further highlights how pressure from school authorities and community may hinder the successful long-term delivery of school-based puberty and menstruation education programs that are external to the national curriculum. In order to feasibly improve menstrual health and hygiene and puberty education in Bangladeshi classrooms in a sustainable way, we recommend a simultaneous three-pronged approach comprising: 1) revision of the current national curriculum to incorporate more comprehensive puberty and menstruation information including its physiology, management, and social context, 2) adequate training and support for teachers to deliver the content, and 3) incorporation of puberty and menstruation content onto students’ national examinations which may better ensure teachers are given the tools and opportunity to prioritize teaching this content.

## Paper context

In a 2014 Bangladesh national survey, 41% of girls reported missing school during their menstrual periods. Despite successful government and non-government menstruation education programs, sustainability beyond short project implementation periods is limited. Our study explores schoolteachers’ perspectives on the factors that prevent continuation and practical approaches to ensure sustainability of puberty and menstruation education in urban and rural Bangladeshi schools.
